# Hydrogen disorder in kaatialaite Fe[AsO_2_(OH)_2_]5H_2_O from Jáchymov, Czech Republic: determination from low-temperature 3D electron diffraction

**DOI:** 10.1107/S2052252520015626

**Published:** 2021-01-01

**Authors:** Gwladys Steciuk, Juraj Majzlan, Jakub Plášil

**Affiliations:** a Department of Structure Analysis, Institute of Physics, Czech Academy of Sciences, Na Slovance 1999/2, Prague 8, 182 21, Czech Republic; b Institute of Geosciences, Friedrich-Schiller University, Burgweg 11, Jena, 07749, Germany

**Keywords:** 3D electron diffraction, kaatialaite, ferric arsenate, hydrogen bonds, disorder

## Abstract

Kaatialaite mineral Fe[AsO_2_(OH)_2_]5H_2_O from Jáchymov, Czech Republic forms white aggregates of needle-shaped crystals with micrometric size. Its structure at ambient temperature has already been reported but hydrogen atoms could not be identified from single-crystal X-ray diffraction. An analysis using 3D electron diffraction at low temperature brings to light the hydrogen positions and the existence of hydrogen disorder. At 100 K, kaatialaite is described in a monoclinic unit cell of *a* = 15.46, *b* = 19.996, *c* = 4.808 Å, β = 91.64° and *V* = 1485.64 Å^3^ with space group *P*2_1_/*n*. The hydrogen sites were revealed after refinements both considering the dynamical effects and ignoring them. The possibility to access most of the hydrogen positions, including partially occupied ones among heavy atoms, from the kinematical refinement is due to the recent developments in the analysis of 3D electron data. The hydrogen bonding observed in kaatialaite provides examples of H_2_O configurations that have not been observed before in the structures of oxysalts with the presence of unusual inverse transformer H_2_O groups.

## Abstract 2

The hydrogen disorder in kaatialaite mineral Fe[AsO_2_(OH)_2_]5H_2_O from Jáchymov, Czech Republic, is reported. A new analysis using 3D electron diffraction at low temperature demonstrates that even partial hydrogen positions are accessible among heavy atoms from the refinement of 3D electron-diffraction data, even when ignoring the dynamical effects.

## 1. Introduction   

Kaatialaite is an environmentally crucial acidic ferric arsenate with the formula Fe^III^[AsO_2_(OH)_2_]_3_(H_2_O)_*n*_, where *n* ranges from 3 to 5 (Boudjada & Guitel, 1981 ▸; Raade *et al.*, 1984 ▸). Kaatialaite has a highly acidic pH (below 2) and forms during the decomposition of primary Fe–As minerals, such as arsenopyrite, löllingite or native arsenic under oxidizing conditions. It has been reported to form directly from aqueous solutions with colossal concentrations of arsenic (the equivalent of ∼10% H_3_AsO_4_) in Jáchymov, Czech Republic (Majzlan, Drahota *et al.*, 2014 ▸; Majzlan, Plášil *et al.*, 2014 ▸) and has been documented from waste piles of arsenopyrite–löllingite concentrate in Přebuz, Czech Republic (Filippi, 2004 ▸). The structure of synthetic kaatialaite has been described by Boudjada & Guitel (1981 ▸); nevertheless, the positions of the hydrogen atoms remained undetected from X-ray single-crystal data.

The current study aims to contribute to the knowledge of this mineral by revealing the positions of hydrogen atoms and clarifying the real content of the molecular H_2_O in its structure through employing a precession-assisted 3D electron diffraction (3D ED) method under cryogenic conditions (Gemmi *et al.*, 2019 ▸). The possibility of revealing the positions of hydrogen atoms from a difference potential map after dynamical refinement has already been shown for the first time for several organic and inorganic compounds (Palatinus *et al.*, 2017 ▸). Later on, Brázda *et al.* documented how to access unambiguously the absolute configuration of crystals containing only light atoms from the dynamical refinement of 3D ED data (Brázda *et al.*, 2019 ▸). Fine structural details are now accessible thanks to the progress in data processing of 3D ED data (Palatinus *et al.*, 2019 ▸). They include the possibility to fit the reflections profile (intensity as a function of the excitation error for several resolution ranges) leading to more accurate integration of the intensities together with the scaling of the frames (for the structure solution and kinematical refinement), and the correction of the frame orientation (for both kinematical and dynamical refinements). These recent advances lead to more suitable data for subsequent structure determination and refinement. Most of the works published were carried out on organic samples where dynamical diffraction effects are weaker because of the presence of atoms having weak scattering power. However, with the example of the mineral vyacheslavite, U(PO_4_)(OH), the dynamical refinement proved its efficiency in determining hydrogen positions, even among the heaviest atoms like uranium (Steciuk *et al.*, 2019 ▸). The current implementation of the dynamical theory in refining the structure leads to very good accuracy but it assumes perfect crystallinity that is often not the case for natural minerals. Moreover, because of computational cost, this approach is still limited to rather small unit cells with a limited number of parameters and the refinement can become a burden when several data sets have to be combined (Jansen *et al.*, 1998 ▸; Palatinus, Corrêa *et al.*, 2015 ▸; Palatinus *et al.*, 2017 ▸; Brázda *et al.*, 2019 ▸). More recently, hydrogen positions could also be determined and refined for organic pharmaceuticals, even ignoring the effects of dynamical scattering during the refinement (kinematical approximation), from continuous-rotation electron data with the advantages of a highly sensitive hybrid pixel detector (Clabbers *et al.*, 2018 ▸, 2019 ▸). In the present work, the refinements both considering the dynamical scattering and ignoring it from precession-assisted 3D ED data bring to light the hydrogen disorder present in kaatialaite, Fe(H_2_AsO_4_)_3_5(H_2_O). More interestingly, most of the hydrogen positions are already accessible from the fast kinematical refinement using data of reasonably good quality but not of very high quality. The kinematical refinement proves its efficiency to get insights into the hydrogen network in inorganic structures containing heavy atoms that would not meet the criteria for the dynamical refinement, like is usually the case for natural supergene minerals.

## 2. Method   

### 2.1. Electron-diffraction data acquisition   

**Figure 1:**
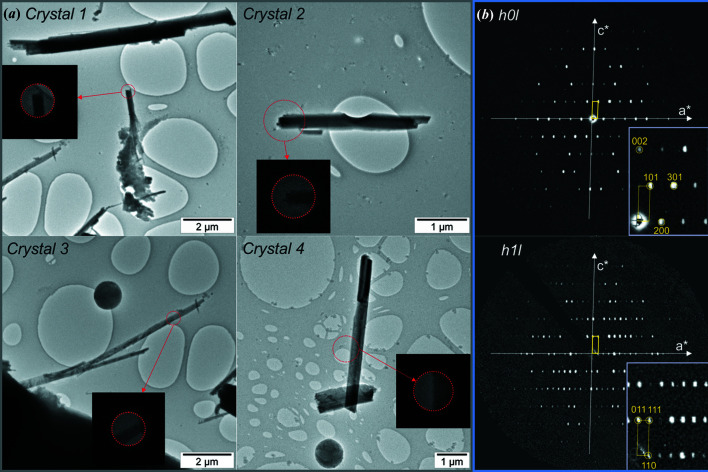
(*a*) Crystals of kaatialaite selected for 3D ED data acquisitions. (*b*) Sections of the reciprocal space from the 3D ED data showing the presence of the *n*-glide plane perpendicular to b*. The unit cell is represented in yellow.

Kaatialaite from Jáchymov forms white aggregates of needle-shaped crystals with micrometric size. The mineral is a good candidate for transmission-electron-microscopy analysis as the section of the needles is below 1 µm (Fig. 1 ▸). For transmission-electron-microscopy analysis, the mineral was gently crushed in acetone and deposited on an Au grid with a thin film of holey amorphous carbon. In order to preserve the hydrated structure of the mineral under vacuum, the grid was plunged into liquid nitro­gen and transferred to an FEI Tecnai 02 transmission electron microscope (TEM) (with an acceleration voltage of 200 kV, LaB_6_) using a Gatan cryo-transfer holder. The precession electron diffraction (PEDT) method was used to collect 3D ED data sets (Kolb *et al.*, 2007 ▸; Mugnaioli *et al.*, 2009 ▸; Vincent & Midgley, 1994 ▸). Several data sets were recorded at 100 K on different crystals with a Nanomegas Digistar precession device and an Olympus Veleta side-mounted CCD camera with an Octane 14 bit dynamic range energy-dispersive analyser silicon drift detector from EDAX (Fig. 1 ▸). The precession angle was set to 1° with a tilt step of 1°. To reduce the electron dose, a condenser aperture of 10 µm and a low illumination setting (spot size 7 or 8) were used.

### 2.2. Data processing   

**Figure 2:**
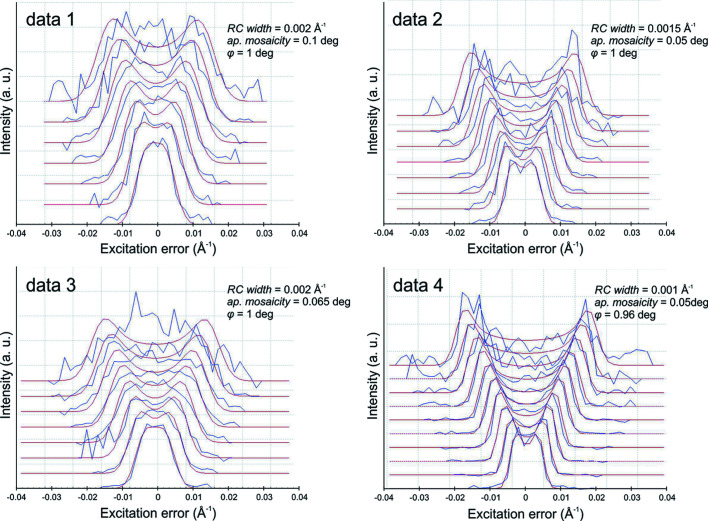
Plots of the RC profiles of the experimental PEDT data collected on the four crystals of kaatialaite used in the refinements (kinematical and dynamical). The lowest blue curve is the averaged RC in the range 0.2 to 0.3 Å^−1^ and the next curves are obtained by steps of 0.1 Å^−1^.

Precession ED data were analyzed using the computer programs *PETS*2.0 (Palatinus *et al.*, 2019 ▸), *Superflip* (Palatinus & Chapuis, 2007 ▸; Palatinus, 2013 ▸) and *Jana2006* (Petříček *et al.*, 2014 ▸). The monoclinic unit-cell dimensions are in line with those reported by Boudjada & Guitel (1981 ▸): *a* = 15.460 Å, *b* = 19.996 Å, *c* = 4.808 Å, β = 91.64° and *V* = 1485.64 Å^3^. At low temperature, the volume is ∼5% higher than at the ambient temperature from X-ray data. The negative thermal expansion is easily explained by the high water content in the structure of kaatialaite. At 100 K, the reciprocal-space sections indicate a space group of *P*2_1_/*n* (with the condition *h* + *l* = 2*n* on *h*0*l*). A way to evaluate the quality of ED tomography data is to generate a rocking curve (RC or camel plot) for each data set. The crystal-related variables influencing the RC profile are reflection full width at half-maximum (RC width) and the apparent mosaicity (for more information, see Brázda *et al.*, 2019 ▸). A good fit between the experimental RC and the calculated RC can be key to the structure solution and to improving the refinement both kinematically and dynamically (Brázda *et al.*, 2019 ▸). When no strong mosaicity or disordering features are visible in the data, a poorly defined RC can be a result of the non-accurate orientation of some frames in the data set. This can influence the *ab initio* structure determination as well as the refinement because the reflections are not properly integrated. For the kaatialaite data, the optimization of the frame orientation in *PETS*2.0 significantly improved the data reduction illustrated with a better match between the simulated and the experimental RC and the improvement of the *R*_int_ values of ∼2.5%. For data 2 and 4, the RC width is found between 0.001 and 0.0015 Å^−1^, and the apparent mosaicity is kept at the default value of 0.05°. These two camel plots present a sharp double hump characteristic of precession ED. The RC plots for data 1 and 3 are slightly less sharp owing to stronger crystal mosaicity (RC width = 0.002 Å^−1^ and apparent mosaicity = 0.1° and 0.065°, respectively) (Fig. 2 ▸). For each data set, two *hkl*-type files are obtained: one file for the structure solution and the kinematical refinement, and another file dedicated to the dynamical refinement where each ED frame is considered independent (Palatinus, Petříček *et al.*, 2015 ▸; Palatinus, Corrêa *et al.*, 2015 ▸). Four data sets were combined to increase the data completeness up to 93% for a sin θ/λ = 0.7 Å^–1^ resolution shell (Table 1 ▸). A complete structure solution similar to the synthetic crystal structure of Fe(H_2_AsO_4_)_3_5(H_2_O) was obtained in the space group *P*2_1_/*n* (Boudjada & Guitel, 1981 ▸). It contains three independent As atoms, one Fe atom, 12 O atoms involved in the coordination polyhedra, and five non-bonded water–oxygen atoms. Fe and As are present in octahedral and tetrahedral coordinations, respectively.

### 2.3. Structure refinement   

**Figure 3:**
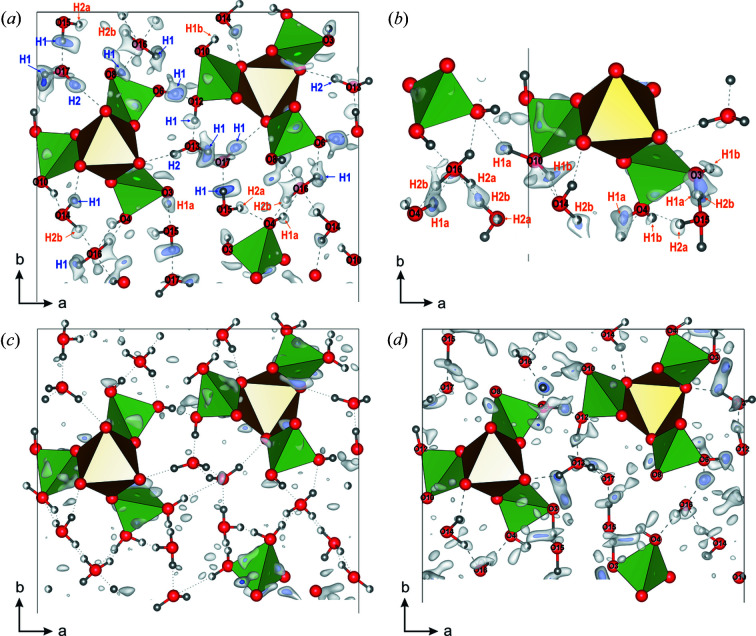
(*a*) Crystal structure viewed along the *c* axis with the superimposed difference Fourier map represented as isosurfaces 2σ [Δ*V*(*r*)] (white) and 3σ [Δ*V*(*r*)] (purple) with visible hydrogen positions after the dynamical refinement. Residual potential and structure obtained after the dynamical refinement of the structure including (*b*) only the non-disordered hydrogen sites (blue) and (*c*) with all hydrogen sites. (*d*) Crystal structure viewed along [001] with the superimposed difference Fourier map from the kinematical refinement.

**Figure 4:**
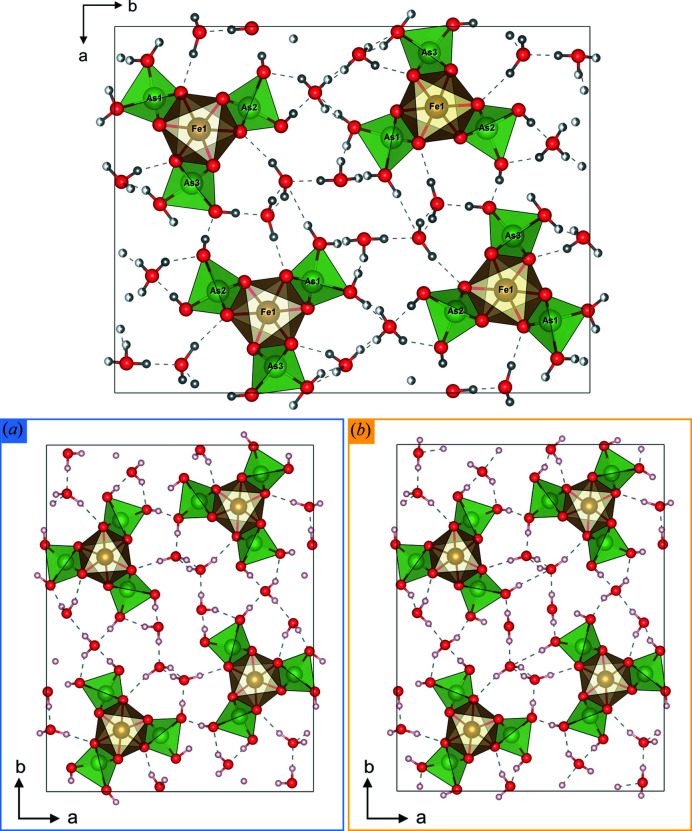
Representation along [001] of the kaatialaite structure obtained from the dynamical refinement with the two hydrogen configurations (*a*) and (*b*).

In order to determine the hydrogen positions, the structure solution was followed by the refinement. Two refinement procedures were carried out against PEDT data: kinematical refinement where the integrated intensities are assumed to be proportional to the square of the structure-factor amplitude, fast but with questionable accuracy, and accurate but time-consuming dynamical refinement where multiple scattering is considered (Palatinus, Petříček *et al.*, 2015 ▸; Palatinus, Corrêa *et al.*, 2015 ▸). For kaatialaite, combining several data sets was not necessary for the *ab initio* structure solution, but for the refinement, combining four data sets was a way to enhance the signal from the hydrogen atoms as well as reducing the noise (Palatinus *et al.*, 2017 ▸). The structure refinement was initially carried out using programs *Jana2006* and *Dyngo* with all non-hydrogen atoms. The first step of the dynamical refinement gives *R*(obs)/*wR*(obs) = 12.23%/11.57% for 7898 observed reflections and 84 structural parameters to refine (Table 1 ▸). According to the stoichiometry of the structure and the bond-valence considerations assuming As^5+^ and Fe^3+^, 6 OH hydroxyl groups are expected for oxygen atoms with bond valences (O*i*) from 1.171 (11) vu to 1.342 (19) vu (As1 = O3, O4; As2 = O6, O8; and As3 = O10, O12), as well as ten hydrogen atoms for the five H_2_O molecules [O13 to O17 with bond valences (O*i*) from 0.0653 (7) vu to 0.1185 (11) vu]. The dynamical refinement leads to a difference potential map [Δ*V*(*r*), where *V* is electrostatic potential and *r* is the positional vector in the unit cell; see Palatinus *et al.* (2017 ▸)], which allowed localization of hydrogen atoms [Fig. 3 ▸(*a*)]. By comparison with organic materials, the difference potential map appears noisier because heavier atoms are present. In the work of Steciuk *et al.* (2019 ▸), the hydrogen position in the mineral vyacheslavite U(PO_4_)(OH) was visible from the difference potential map after the dynamical refinement despite the presence of quite strong surrounding artifacts, and this position was further confirmed by density-functional-theory calculations. In Fig. 3 ▸, significant maxima in the residual potential map are found at expected hydrogen positions (see Table S1 in the Supporting information). It was assumed that each hydrogen H1O*i* (and H_2_O*i* for water H_2_O) forms a covalent bond with O*i* and is stabilized by a strong hydrogen bond to O*j* (and O*k* for H_2_O*i*). In kaatialaite, two hydrogen configurations [called (*a*) and (*b*)] are needed to explain all of the first 16 observed maxima (Table S1). The standard deviation (σ) of Δ*V*(*r*) is ∼0.2 e Å^–1^; the heights of the maxima corresponding to the fully occupied hydrogen atoms range between 3.6σ and 2.3σ. Surprisingly, six out of the 12 disordered hydrogen positions (with partial occupancies) are well visible with maxima ranging from ∼3.2σ to 2.2σ. In order to confirm the existence of two configurations and detect the missing hydrogen positions, the dynamical refinement was carried out further including the most visible hydrogen sites. The intermediate refinement led to *R*(obs)/*wR*(obs) = 11.90%/11.43%. The new resulting difference potential map in Fig. 3 ▸(*b*) presents slightly sharper maxima related to disordered hydrogen that helps to define the last hydrogen positions. Only one hydrogen site could not be revealed from the dynamical refinement and was set at the most likely position (H_2_O16*a*). After identification of the last hydrogen positions, hydrogen atoms and the structure were refined with distance restraints of 0.98 Å on all O–H distances, and the isotropic displacement parameters of the hydrogen atoms were set as riding with a extension factor of 1.2. One could suggest that the hydrogen disorder comes from the data combination and that the crystals possess either one or the other hydrogen configuration. However, by generating the difference potential map from each data set separately, maxima with significant height from the two configurations confirmed that hydrogen disorder is an intrinsic property of kaatialaite (see Fig. S1 in the Supporting information). After the refinement with all the hydrogen atoms (*R*(obs)/*wR*(obs) = 11.46%/10.90%) and the application of the frame-orientation refinement, which is a part of the standard dynamical refinement procedure, the combined *R* factors decreased to *R*(obs) = 9.90% and *wR*(obs) = 9.19% (Table 1 ▸). The structural parameters of kaatialaite are listed in Table S2 together with the bond valences. The cation–oxygen distances and oxygen–hydrogen distances are summarized in Table S3. The structure in the two configurations is represented in Fig. 4 ▸. The refinement of the water content was not significantly different from *n* = 5 showing that natural kaatialaite from Jáchymov is very close to the synthetic phase described by Boudjada & Guitel (1981 ▸).

For comparison, the kinematical refinement approach was also performed to refined kaatialaite structure. The four data sets were merged and the refinement of the cations and oxygen parameters gives *R*(obs)/*wR*(obs) = 18.60%/17.99% for 1254 observed reflections and 85 refined parameters (Table 1 ▸). In the case of kaatialaite, the Δ*V*(*r*) map obtained from kinematical refinement shows maxima compatible with hydrogen positions [Fig. 3 ▸(*d*)]. Knowing where hydrogen positions are expected from the previous result of the dynamical refinement and symmetry considerations, 13 of the 22 hydrogen sites are visible from the difference potential map, including six positions with isosurface levels above 3σ [Δ*V*(*r*)]. Contrary to the Δ*V*(*r*) from dynamical refinement, the disordered hydrogen positions that are very close to each other tend to give broad and rather diffuse maxima, at an equidistance of the two involved oxygen atoms instead of two distinct maxima. This phenomenon was already observed in the work of Palatinus *et al.* (2017 ▸) for disordered atoms after dynamical refinement. Nevertheless, for kaatialaite, enough hydrogen positions are revealed from the kinematical refinement to assume two hydrogen configurations. The final kinematical refinement, including all hydrogen atoms, improves the *R*-factor values by more than 1% with *R*(obs)/*wR*(obs) = 17.52%/16.91% (Table 1 ▸). The structural parameters are shown in Table S4. Alone, this result may seem questionable because the difference potential maps are noisier when dynamical effects are ignored in the refinement. However, the comparison with the results obtained from the dynamical refinement confirms the efficiency in detecting part of the hydrogen sites, even ignoring dynamical effects in the refinement when data are of sufficient quality without being of very high quality (the lack of resolution in data 1, mosaicity). This result is directly owing to the new implementation in *PETS*2.0, bringing significant improvement to the data reduction of 3D ED data (Palatinus *et al.*, 2019 ▸).

Although the hydrogen disorder in kaatialaite probably results in a non-negligible amount of inelastic scattering influencing the diffracted amplitudes and thus the refinement, the technique used was not appropriate to experimentally collect this information with accuracy. Nevertheless, an estimate of the inelastic scattering can be evaluated through the ratio of the inelastic scattering cross section to the elastic one as σ_i_/σ_e_ ≃ 20/*Z* for a TEM operating at 200 kV, with *Z* being the atomic number of the average atom in kaatialaite (averaged from 21 atoms with one Fe, one As, nine O and ten H) (Latychevskaia & Abrahams, 2019 ▸; Egerton, 2011 ▸). For kaatialaite, this ratio is ∼3:1 meaning that only one scattering event in four is elastic. In our data, this inelastic scattering is not present as diffuse features out of the Bragg spots but only as a likely weak broadening of the Bragg peaks, meaning that no elastic scattering occurs after the inelastic events (see a further explanation in the work of Latychevskaia & Abrahams, 2019 ▸). We assume that the influence on the diffracted intensities remains small because the dynamical refinement that considers the multiple scattering but not the inelastic part leads to very satisfying results (low *R* values and meaningful structural parameters).

## 3. Discussion and conclusions   

**Figure 5:**
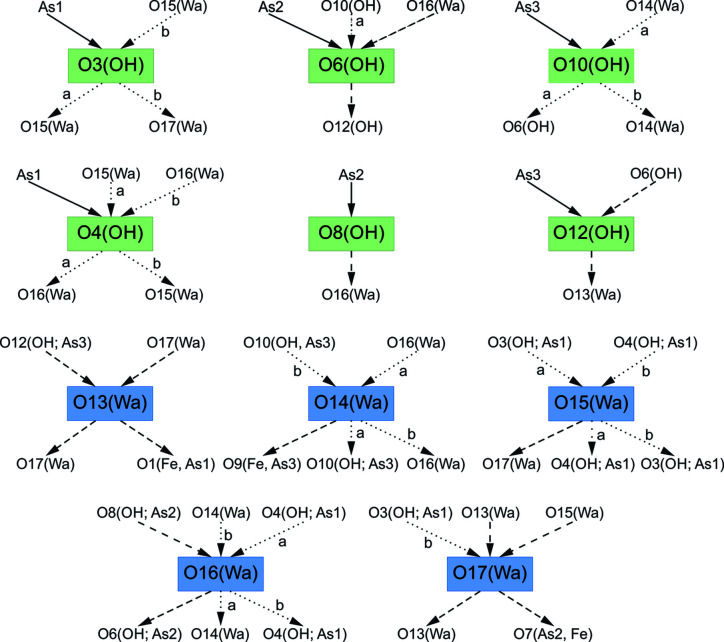
Hydrogen bonding in the structure of kaatialaite. OH (hydroxyl), water (H_2_O) (Wa) group, regular (thick) arrow = strong cation–oxygen bond, dashed arrow = hydrogen bond, and dotted arrow = alternative [depending on the configuration marked either by (*a*) or (*b*)] hydrogen bond.

Regarding water molecules, O13 and O17 keep the same hydrogen environments while the three other water molecules, O14, O15 and O16, exist in two configurations called (*a*) and (*b*). These three disordered water molecules are linked to one non-disordered hydrogen atom (common to both configurations), and the position of the second hydrogen site is split and creates a strong hydrogen bond with two oxygen atoms O*j* and O*k*. Similar behavior is observed for the acid groups: H1O6, H1O8 and H1O12 are not disordered. In contrast, H1O*i* of O3, O4 and O10 are split into two partially occupied positions. In hydrated oxysalts, there are generally several types of H_2_O moieties in their crystal structures, each of them playing a slightly distinct structural role, as reviewed in the works of Hawthorne (1992 ▸), Hawthorne & Sokolova (2012 ▸), Hawthorne & Schindler (2008 ▸) and Schindler & Hawthorne (2008 ▸). Particular types of H_2_O can be distinguished based on the coordination number of O atoms in these (H_2_O) groups. According to the articles mentioned above, in the structures of oxysalts, we distinguish among the transformer, non-transformer and inverse transformer (H_2_O) groups with three, four and fivefold coordinated O atoms, respectively. Their role is generally to transfer the bond valence from cations (Lewis acids) to anions (Lewis bases), keeping the structure together, as the strengths of these components are equal or similarly matching, following the valence-matching principle of the bond valence (Brown, 2002 ▸, 2009 ▸; Hawthorne & Sokolova, 2012 ▸). Our structure refinement has indicated that there is disorder among some of the hydrogen atoms in the structure of kaatialaite. Based on the results of the bond-valence analysis (Tables S2 and S5), we can conclude that both possible configurations of the hydrogen atoms within the structure lead to meaningful and acceptable final bond valences of all anions, giving sums incident upon the O sites of around 2 vu. The coordination environment of all the atomic sites involved in the hydrogen bonding is displayed in Fig. 5 ▸. Generally, the coordination of OH sites related to the double-protonated As tetrahedra is relatively simple. Some of the OH sites occur unchanged in both configurations (O6, O8 and O12 sites) while the remaining three OH sites show two possible configurations (O3, O10 and O4 sites). Regarding hydrogen bonds incident to these OH sites, the situation is more complicated because the corresponding O atoms act as acceptors of the hydrogen bonds from the (H_2_O) groups located in the interlayer. According to our observations, there are two (H_2_O) groups that act as transformer groups with three coordinated atoms, in each possible configuration (O14 and O15) (Fig. 5 ▸). O16 and O13 appear as non-transformers in the two configurations, accepting and giving two bonds, respectively. The last (H_2_O) group, O17, is either non-transformer or inverse-transformer in the second configuration, accepting three bonds and with two weak bonds emanating from it (Fig. 5 ▸). Inverse-transformer groups play a similar role in the structures as transformer groups; nevertheless, they seldom occur in oxysalts (Schindler & Hawthorne, 2008 ▸). Moreover, hydrogen bonding in kaatialaite provides examples of (H_2_O) configurations that have not been observed in the structures of oxysalts before. For instance, one of the possible configurations of O17(water) acts as an inverse-transformer (H_2_O) group [*c.f.* Fig. 5 in the work of Schindler & Hawthorne (2008 ▸); in particular, the coordination labeled as (7)]. The nature of the hydrogen disorder in kaatialaite remains unclear and needs to be further studied. However, it may be, to some extent, dynamical. For several hydrated minerals, static models are insufficient to understand the role and behavior of the H_2_O molecules (Winkler, 1996 ▸). Finally, to be able to investigate the interaction between the H_2_O molecules and their neighbors, complementary techniques should be employed, such as solid-state NMR infrared spectroscopy, proton NMR or incoherent neutron scattering.

Coming back to the structural analysis, the absence of hydrogen in the previously published model, as well as the impossibility to determine it from more recent attempts after X-ray single-crystal measurements, may be explained by the stronger hydrogen disorder (partially occupied sites) at room temperature where the previous X-ray measurements were carried out. For this purpose, low-temperature measurements allow one to lock the structure in a more static state where hydrogen positions become easier to localize. Moreover, ED benefits from the improved contrast of hydrogen atoms (Egerton, 2011 ▸). The enhanced contrast of light atoms relative to heavier atoms implies a more significant contribution from the hydrogen atoms to the overall signal, especially in kaatialaite that is built from a noticeable amount of hydrogenous moieties (Vainshtein, 1964 ▸; Dorset, 1995 ▸). The dynamical refinement again proved its efficiency in detecting hydrogen positions, even those that are partially occupied. However, with 43 atoms overall in the monoclinic unit cell and four data sets to reach a reasonable coverage, the computation time already required several days. For data collected on rather well crystallized sample, the improvement of the data reduction in *PETS*2.0 also allows a better kinematical refinement, illustrated in this study by the possibility of retrieving hydrogen information after the faster kinematical refinement. Although the kinematical approximation does not lead to the same accuracy in terms of structural parameters, it is enough to gain an insight into the hydrogen positions and reveal the existence of hydrogen disorder in the natural kaatialaite (Table S1). In general, whatever 3D ED technique is used (Gemmi & Lanza, 2019 ▸), it represents an interesting point for structures of many natural minerals, especially the minerals coming from second mineralization processes that are not well crystallized and present rather low symmetry. Their structure cannot be refined considering the dynamical theory because of computational burden and their imperfect crystallinity (Shi *et al.*, 2013 ▸; Gemmi *et al.*, 2015 ▸; Nannenga *et al.*, 2014 ▸; Cichocka *et al.*, 2018 ▸). We can include in this category many layered minerals possessing interlayer hydrogen bonding. For these minerals, the localization of hydrogen is key to understanding the stability of the mineral (Conroy *et al.*, 2017 ▸).

## Supplementary Material

Crystal structure: contains datablock(s) global, I. DOI: 10.1107/S2052252520015626/ct5014sup1.cif

Supporting information . DOI: 10.1107/S2052252520015626/ct5014sup2.pdf

CCDC reference: 2047162

## Figures and Tables

**Table 1:** A summary of data-collection conditions and refinement parameters for kaatialaite

Structural formula	Fe(H_2_AsO_4_)_3_ *n*(H_2_O), *n* = 5
Unit–cell parameters (PEDT) 100 K	
*a* (Å)	15.460
*b* (Å)	19.996
*c* (Å)	4.808
α = γ (°)	90
β (°)	91.64
*V* (Å^3^)	1485.64
*Z*	4
Density (g cm^−3^)	2.3457
Space group	*P*2_1_/*n*
Temperature (K)	100
TEM	FEI Tecnai T20
Radiation (wavelength) (Å)	Electrons (0.0251)
Resolution range sin θ/λ ( Å^–1^)	0.05–0.7
Limiting Miller indices	–22 < *h* < 22, 0 < *k* < 26, 0 < *l* < 6
No. of independent reflections (obs/all) – kinematic	1269/4299
*R*_int_ (obs/all) – kinematic (all data combined) (%)	29.63/58.64
Redundancy (all data combined)	8.496
Coverage for sin θ/λ = 0.7 Å^−1^ (all data) (%)	93
	
Kinematical refinement	
No. of reflections (obs/all)	1281/4299
Kinematical refinement without hydrogen	
*R, wR* (obs) (%)	18.78/18.77
*N* parameters	85
Kinematical refinement with hydrogen	
*R, wR* (obs) (%)	17.99/17.74
*N* parameters	151
	
Dynamical refinement	
RSg(max)	0.6
Crystal thickness (crystals 1 to 4) (Å)	641, 795, 464, 1129
Without hydrogen and no optimization of the frames’ orientation	
No. of reflections (obs/all)	7898/59148
*R, wR* (obs) (%)	12.23/11.57
*N* parameters/*N* structural parameters	461/84
With hydrogen and no optimization of the frames’ orientation	
No. of reflections (obs/all)	7898/59148
*R, wR* (obs) (%)	11.46/10.90
*N* parameters/*N* structural parameters	527/150
With hydrogen and optimization of the frames’ orientation	
No. of reflections (obs/all)	7661/56238
*R, wR* (obs) (%)	9.90/9.19
*N* parameters/*N* structural parameters	509/150
