# Supplementary material for: Hydrogen disorder in kaatialaite Fe[AsO2(OH)2]5H2O from Jáchymov, Czech Republic: determination from low-temperature 3D electron diffraction
Source: IUCrJ. 2021 Jan 1;8(Pt 1):116–23. doi: 10.1107/S2052252520015626 (PMC7793002; doi:10.1107/S2052252520015626)
Supplement: Supplementary file 2 [file m-08-00116-sup2.pdf]

**IUCrJ**

**Volume 8 (2021)**

**Supporting information for article:**

## Hydrogen disorder in kaatialaite $Fe[AsO_2(OH)_2]5H_2O$ from Jáchymov, Czech Republic: determination from low-temperature 3D electron diffraction

**Gwladys Steciuk, Juraj Majzlan and Jakub Plášil**

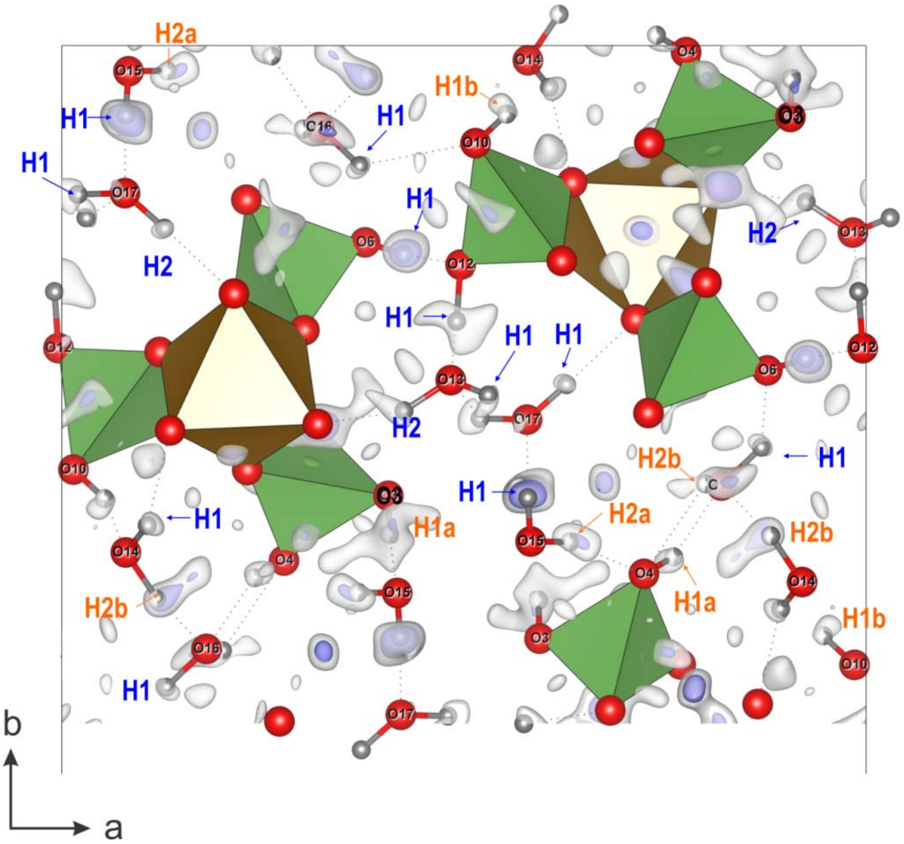


**Figure S1** Crystal structure viewed along axis *c* with the superimposed difference-Fourier map generated from the second data set only and represented as isosurfaces 2σ[Δ*V*(r)] (white) and 3σ[Δ*V*(r)] (blue) with visible hydrogen positions after the dynamical refinement of kaatialaite structure without hydrogen.

**Table S1** Summary of the hydrogen positions revealed from the dynamical and the kinematical refinements. The significance of the maxima in the difference potential map Δ*V*(r) is expressed with the isosurface level σ.

| Refinement: | Dynamical | | Kinematical | |
|---|---|---|---|---|
| *Configuration:* | *a* | *b* | *a* | *b* |
| ***OH: H1Oi*** | | | | |
| H1O3 | 3.02σ | 2.81σ* | ≥2σ | ≥2σ |
| H1O4 | 2.50σ | 2.15σ* | ≥2σ | ≥3σ |
| H1O6 | -------------- 3.55σ -------------- | | -------------- ≥3σ -------------- | |
| H1O8 | -------------- 2.85σ -------------- | | ---------- not visible --------- | |
| H1O10 | 3σ* | 2σ | not visible | ≥2σ |
| H1O12 | -------------- 3.02σ -------------- | | -------------- ≥3σ -------------- | |
| **$H_2O$: *H1Oi/ H2Oi*** | | | | |
| H1O13/H2O13 | ----------- 3.38σ / 2.31σ ----------- | | --------- ≥3σ / ≥3σ --------- | |
| H1O14/H2O14 | 3.55σ / 2σ* | 3.55σ / 2.50σ | ≥2σ / not visible | ≥2σ / ≥2σ |
| H1O15/H2O15 | 3.55σ / 2.50σ | 3.55σ / 2.80σ* | ≥2σ / not visible | ≥2σ / not visible |
| H1O16/H2O16 | 3.38σ / not visible | 3.38σ / 3.20σ | ≥3σ / not visible | ≥3σ / ≥2σ |
| H1O17/H2O17 | ----------- 3.20σ / 3.20σ ----------- | | 3σ / not visible | |

* the heights of the maxima corresponding to the hydrogen atoms obtained after a second step of dynamical refinement involving the first visible hydrogen.

**Table S2** Atom positions, displacement parameters (Å2) and bond-valence sums (BVS, in valence-units) for kaatialaite as obtained from dynamical refinement.

| *atom* | *occu* | *x/a* | *y/b* | *z/c* | *Uiso* | *BVS* |
|---|---|---|---|---|---|---|
| As1 | 1 | 0.19667(7) | 0.08330(8) | 0.1282(2) | 0.0121(3) | 4.84(3) |
| As2 | 1 | 0.22229(7) | 0.28286(8) | 0.1154(2) | 0.0103(3) | 5.12(3) |
| As3 | 1 | 0.42806(7) | 0.16204(8) | 0.1411(2) | 0.0127(3) | 4.83(3) |
| Fe1 | 1 | 0.28056(7) | 0.17728(9) | 0.6325(2) | 0.0072(3) | 2.752(11) |
| O1 | 1 | 0.18162(18) | 0.1392(2) | 0.3758(6) | 0.0218(9) | 1.922(16) |
| O2 | 1 | 0.2682(2) | 0.0941(2) | -0.1187(6) | 0.0203(9) | 1.841(13) |
| O3 | 1 | 0.0937(2) | 0.0687(2) | -0.0095(7) | 0.0304(11) | 2.10(6) |
| O4 | 1 | 0.22467(19) | 0.0046(2) | 0.2706(6) | 0.0201(10) | 1.98(5) |
| O5 | 1 | 0.1974(2) | 0.2272(3) | -0.1229(6) | 0.0258(10) | 1.97(2) |
| O6 | 1 | 0.1247(2) | 0.3134(2) | 0.2395(6) | 0.0314(10) | 2.04(3) |
| O7 | 1 | 0.28702(19) | 0.2637(2) | 0.3902(6) | 0.0208(9) | 1.79(1) |
| O8 | 1 | 0.2721(2) | 0.3499(3) | -0.0098(7) | 0.0399(12) | 2.22(4) |
| O9 | 1 | 0.3661(2) | 0.1287(2) | 0.3878(6) | 0.0219(9) | 1.88(1) |
| O10 | 1 | 0.4848(2) | 0.0954(3) | 0.0130(8) | 0.0438(13) | 2.15(7) |
| O11 | 1 | 0.37989(18) | 0.2074(2) | -0.1103(6) | 0.0178(9) | 1.84(1) |
| O12 | 1 | 0.5051(2) | 0.2118(2) | 0.3087(6) | 0.0297(10) | 2.11(3) |
| O13 | 1 | 0.51566(19) | 0.3215(2) | 0.0500(5) | 0.0391(12) | 2.04(3) |
| O14 | 1 | 0.9241(2) | 0.4860(2) | 0.1268(7) | 0.0326(11) | 1.86(5) |
| O15 | 1 | 0.9193(3) | 0.0246(2) | 0.3880(6) | 0.0429(12) | 1.88(8) |
| O16 | 1 | 0.6806(2) | 0.0784(3) | 0.0903(7) | 0.0420(12) | 1.98(4) |
| O17 | 1 | 0.9232(2) | 0.1404(2) | 0.0636(5) | 0.0402(12) | 1.97(4) |
| H1o3a | 0.5 | 0.093(2) | 0.0317(12) | -0.144(6) | 0.036456 | 1.07(7) |
| H1o3b | 0.5 | 0.0434(13) | 0.0937(19) | 0.055(5) | 0.036456 | 0.97(5) |
| H1o4a | 0.5 | 0.261(2) | 0.9698(11) | 0.189(3) | 0.024118 | 0.98(5) |
| H1o4b | 0.5 | 0.8258(14) | 0.0121(13) | 0.631(5) | 0.024118 | 0.99(5) |
| H1o6 | 1 | 0.0808(8) | 0.3096(11) | 0.089(2) | 0.037707 | 1.01(3) |
| H1o8 | 1 | 0.2378(11) | 0.3758(11) | -0.147(3) | 0.047882 | 1.01(4) |
| H1o10a | 0.5 | 0.534(2) | 0.124(2) | 0.965(4) | 0.052573 | 0.97(6) |
| H1o10b | 0.5 | 0.4470(14) | 0.066(2) | 0.901(6) | 0.052573 | 1.04(6) |
| H1o12 | 1 | 0.5091(11) | 0.2542(5) | 0.207(4) | 0.035683 | 1.10(3) |
| H1o13 | 1 | 0.4754(6) | 0.3315(12) | -0.107(2) | 0.046971 | 0.99(2) |
| H2o13 | 1 | 0.5745(4) | 0.3345(12) | 0.001(3) | 0.046971 | 0.99(2) |
| H1o14 | 1 | 0.8938(11) | 0.4520(4) | 0.013(3) | 0.039105 | 1.03(3) |
| H2o14a | 0.5 | 0.9426(16) | 0.4664(6) | 0.3061(19) | 0.039105 | 1.06(5) |
| H2o14b | 0.5 | 0.6183(11) | 0.0169(12) | 0.297(6) | 0.039105 | 0.99(5) |
| H1o15 | 1 | 0.9207(11) | 0.0667(5) | 0.283(3) | 0.051441 | 0.99(2) |
| H2o15a | 0.5 | 0.8685(14) | 0.0242(9) | 0.506(6) | 0.051441 | 0.97(8) |
| H2o15b | 0.5 | 0.086(3) | 0.0130(6) | -0.258(3) | 0.051441 | 1.05(3) |
| H1o16 | 1 | 0.6428(8) | 0.1064(7) | 0.971(3) | 0.050432 | 0.97(3) |

| | | | | | | |
|---|---|---|---|---|---|---|
| H2o16a | 0.5 | 0.6450(8) | 0.0514(9) | 0.213(3) | 0.050432 | 0.98(3) |
| H2o16b | 0.5 | 0.2750(8) | 0.9425(9) | 0.023(3) | 0.050432 | 0.96(3) |
| H1o17 | 1 | 0.9642(8) | 0.1427(9) | -0.088(2) | 0.048273 | 0.96(2) |
| H2o17 | 1 | 0.8724(8) | 0.1681(8) | 0.017(3) | 0.048273 | 0.97(3) |

**Table S3** Selected interatomic distances (in Å) and angles (degree) in the structure of kaatialaite.

| As-O | | *M*-($H_2O$) | | *M*-OH | |
|---|---|---|---|---|---|
| As1-O1 | 1.653(4) | O13-H1O13 | 0.981(11) | O3-H1O3a | 0.98(3) |
| As1-O2 | 1.658(3) | O13-H2O13 | 0.980(10) | O3-H1O3b | 0.98(3) |
| As1-O3 | 1.728(4) | O14-H1O14 | 0.982(12) | O4-H1O4a | 0.98(3) |
| As1-O4 | 1.766(5) | O14-H2O14a | 0.981(12) | O4-H1O4b | 0.98(2) |
| As2-O5 | 1.634(4) | O14-H2O14b | 0.98(2) | O6-H1O3 | 0.981(13) |
| As2-O6 | 1.746(4) | O15-H1O15 | 0.981(12) | O8-H1O4 | 0.982(18) |
| As2-O7 | 1.675(3) | O15-H2O15a | 0.98(2) | O10-H1O10a | 0.98(4) |
| As2-O8 | 1.666(5) | O15-H2O15b | 0.981(14) | O10-H1O10b | 0.98(3) |
| As3-O9 | 1.683(3) | O16-H1O16 | 0.981(14) | O12-H1O4 | 0.984(13) |
| As3-O10 | 1.719(5) | O16-H2O16a | 0.981(16) | angle $H1O_i$-$O_i$-$H2O_{i(a,b)}$ [°] | |
| As3-O11 | 1.666(4) | O16-H2O16b | 0.981(15) | O13 | 109.5(11) |
| As3-O12 | 1.730(4) | O17-H1O17 | 0.981(12) | O14 conf. a | 109.5(11) |
| Fe1-O1 | 2.078(3) | O17-H2O17 | 0.981(14) | O14 conf. b | 109.5(15) |
| Fe1-O2 | 2.060(4) | | | O15 conf. a | 109.5(15) |
| Fe1-O5 | 2.028(4) | | | O15 conf. b | 109.5(12) |
| Fe1-O7 | 2.088(4) | | | O16 conf. a | 109.5(12) |
| Fe1-O9 | 2.040(3) | | | O16 conf. b | 109.5(13) |
| Fe1-O11 | 2.030(3) | | | O17 | 109.5(12) |
| Strong hydrogen bonds O---H | | | | | |
| O1-H2O13 | 1.853(11) | O10-H2O14a | 1.703(14) | O15-H1O3a | 1.63(3) |
| O3-H2O15b | 1.633(14) | O12-H1O6 | 1.805(13) | O16-H1O8 | 1.771(18) |
| O4-H2O16b | 1.902(17) | O13-H1O12 | 1.546(13) | O16-H2O14b | 1.87(2) |
| O4-H2O15a | 1.91(2) | O13-H1O17 | 2.060(13) | O16-H1O4a | 1.90(2) |
| O6-H1O16 | 1.966(15) | O14-H1O10b | 1.70(3) | O17-H1O13 | 1.843(13) |
| O7-H2O17 | 1.980(15) | O14-H2O16a | 1.868(16) | O17-H1O15 | 1.812(12) |
| O9-H1O14 | 1.770(10) | O15-H1O4b | 1.90(2) | O17-H1O3b | 2.08(3) |

**Table S4** Atom positions, displacement parameters (Å$^2$) for kaatialaite as obtained from kinematical refinement. The value d(A*kin*-A*dyn*) (Å) is the distance between the atoms of this model and the one after dynamical refinement.

| *atom* | *occu* | *x/a* | *y/b* | *z/c* | *Uiso* | *d*($Ai^{kin}$-$Ai^{dyn}$) |
|---|---|---|---|---|---|---|
| As1 | 1 | 0.1965(2) | 0.0845(3) | 0.1255(7) | 0.0186(11) | 0.027(5) |
| As2 | 1 | 0.2215(2) | 0.2827(3) | 0.1162(7) | 0.0204(11) | 0.014(4) |
| As3 | 1 | 0.4275(2) | 0.1613(3) | 0.1438(6) | 0.0181(11) | 0.021(5) |
| Fe1 | 1 | 0.2811(2) | 0.1778(3) | 0.6340(7) | 0.0170(11) | 0.016(5) |

| | | | | | | |
|---|---|---|---|---|---|---|
| O1 | 1 | 0.1793(6) | 0.1383(8) | 0.3832(19) | 0.025(3) | 0.054(11) |
| O2 | 1 | 0.2718(7) | 0.0927(8) | -0.115(2) | 0.028(3) | 0.065(12) |
| O3 | 1 | 0.0897(10) | 0.0703(11) | -0.009(3) | 0.067(5) | 0.069(17) |
| O4 | 1 | 0.2226(7) | 0.0026(9) | 0.256(2) | 0.041(4) | 0.084(13) |
| O5 | 1 | 0.1929(7) | 0.2257(8) | -0.1240(19) | 0.028(3) | 0.076(12) |
| O6 | 1 | 0.1200(6) | 0.3122(7) | 0.2377(18) | 0.020(3) | 0.077(10) |
| O7 | 1 | 0.2926(6) | 0.2648(7) | 0.3889(18) | 0.016(3) | 0.090(10) |
| O8 | 1 | 0.2783(8) | 0.3523(9) | -0.021(2) | 0.048(4) | 0.122(14) |
| O9 | 1 | 0.3637(7) | 0.1300(7) | 0.4017(19) | 0.026(3) | 0.082(11) |
| O10 | 1 | 0.4859(8) | 0.0956(9) | 0.009(2) | 0.050(4) | 0.026(13) |
| O11 | 1 | 0.3808(6) | 0.2049(8) | -0.1145(18) | 0.024(3) | 0.055(15) |
| O12 | 1 | 0.5032(8) | 0.2126(7) | 0.310(2) | 0.037(3) | 0.035(13) |
| O13 | 1 | 0.5160(8) | 0.3211(8) | 0.044(2) | 0.050(4) | 0.030(10) |
| O14 | 1 | 0.9212(8) | 0.4866(6) | 0.124(2) | 0.041(4) | 0.048(12) |
| O15 | 1 | 0.9206(7) | 0.0224(7) | 0.388(2) | 0.031(3) | 0.048(14) |
| O16 | 1 | 0.6807(7) | 0.0800(7) | 0.095(2) | 0.028(3) | 0.037(14) |
| O17 | 1 | 0.9228(8) | 0.1378(8) | 0.063(2) | 0.058(4) | 0.051(17) |
| H1o3a | 0.5 | 0.096(8) | 0.033(5) | -0.14(2) | 0.080268 | 0.06(12) |
| H1o3b | 0.5 | 0.038(6) | 0.094(9) | 0.054(17) | 0.080268 | 0.08(9) |
| H1o4a | 0.5 | 0.242(6) | 0.979(6) | 0.091(10) | 0.048875 | 0.58(7) |
| H1o4b | 0.5 | 0.828(5) | 0.002(6) | 0.63(2) | 0.048875 | 0.20(12) |
| H1o6 | 1 | 0.0663(14) | 0.308(4) | 0.124(6) | 0.024503 | 0.29(3) |
| H1o8 | 1 | 0.235(4) | 0.380(3) | -0.120(7) | 0.057961 | 0.17(5) |
| H1o10a | 0.5 | 0.535(5) | 0.122(6) | 0.94(2) | 0.059886 | 0.12(11) |
| H1o10b | 0.5 | 0.450(5) | 0.073(5) | 0.86(2) | 0.059886 | 0.23(11) |
| H1o12 | 1 | 0.509(4) | 0.2573(15) | 0.226(11) | 0.044726 | 0.11(5) |
| H1o13 | 1 | 0.478(3) | 0.325(3) | -0.124(8) | 0.059873 | 0.16(5) |
| H2o13 | 1 | 0.5699(16) | 0.346(2) | 0.018(8) | 0.059873 | 0.26(5) |
| H1o14 | 1 | 0.895(3) | 0.4521(12) | 0.001(8) | 0.049495 | 0.06(4) |
| H2o14a | 0.5 | 0.945(7) | 0.4655(19) | 0.294(11) | 0.049495 | 0.07(7) |
| H2o14b | 0.5 | 0.624(4) | 0.017(4) | 0.315(17) | 0.049495 | 0.12(7) |
| H1o15 | 1 | 0.916(4) | 0.0655(14) | 0.290(10) | 0.036933 | 0.08(6) |
| H2o15a | 0.5 | 0.867(3) | 0.014(3) | 0.487(13) | 0.036933 | 0.22(7) |
| H2o15b | 0.5 | 0.072(5) | 0.014(2) | -0.251(10) | 0.036933 | 0.22(9) |
| H1o16 | 1 | 0.647(3) | 0.114(2) | 0.988(10) | 0.033437 | 0.18(4) |
| H2o16a | 0.5 | 0.642(3) | 0.056(2) | 0.220(10) | 0.033437 | 0.11(4) |
| H2o16b | 0.5 | 0.302(3) | 0.9559(19) | 0.031(10) | 0.033437 | 0.49(4) |
| H1o17 | 1 | 0.961(2) | 0.145(4) | -0.094(8) | 0.069999 | 0.07(6) |
| H2o17 | 1 | 0.8661(17) | 0.158(2) | 0.022(10) | 0.069999 | 0.22(4) |

**Table S5** Results of the bond-valence analysis for kaatialaite (values given in valence units).

| | O1 | O2 | O3 | O4 | O5 | O6 | O7 | O8 | O9 | O10 | O11 | O12 | O13 | O14 | O15 | O16 | O17 | ΣBV |
|---|---|---|---|---|---|---|---|---|---|---|---|---|---|---|---|---|---|---|
| Fe1 | 0.43 | 0.45 | | | 0.49 | | 0.42 | | 0.47 | | 0.49 | | | | | | | 2.75 |
| As1 | 1.37 | 1.36 | 1.11 | 1.00 | | | | | | | | | | | | | | 4.84 |
| As2 | | | | | 1.45 | 1.06 | 1.29 | 1.32 | | | | | | | | | | 5.12 |
| As3 | | | | | | | | | 1.26 | 1.14 | 1.32 | 1.11 | | | | | | 4.83 |
| H1O3a | | 0.01 | 0.86 | 0.01 | | | | | | | | | | | 0.19 | | | 1.07 |
| H1O3b | 0.01 | | 0.86 | | | | | | | | | | 0.02 | | 0.01 | | 0.07 | 0.97 |
| H1O4a | | 0.01 | | 0.86 | | | | 0.01 | | | | | | | | 0.10 | | 0.98 |
| H1O4b | | | 0.02 | 0.86 | | | | 0.01 | | | | | | | 0.10 | | | 0.99 |
| H1O6 | | | | | 0.02 | 0.86 | | | | | | 0.13 | | | | | | 1.01 |
| H1O8 | | | | | | 0.01 | | 0.86 | | | | | | | | 0.14 | | 1.01 |
| H1O10a | | | | | | 0.05 | | | | 0.86 | 0.01 | 0.03 | | | | 0.02 | | 0.97 |
| H1O10b | | 0.012 | | | | | | 0.008 | | 0.86 | | | | 0.16 | | | | 1.04 |
| H1O12 | | | | | | | | | | | 0.01 | 0.86 | 0.23 | | | | | 1.10 |
| H1O13 | | | | | | | | | | | 0.01 | | 0.86 | | | | 0.12 | 0.99 |
| H2O13 | 0.12 | | | | 0.01 | | | | | | | | 0.86 | | | | | 0.99 |
| H1O14 | | 0.01 | | 0.01 | | | | | 0.13 | 0.02 | | | | 0.86 | | | | 1.03 |
| H2O14a | | 0.01 | | | | | | | 0.01 | 0.17 | | | | 0.86 | 0.01 | | | 1.06 |
| H2O14b | | 0.01 | | | | | | | | 0.01 | | | | 0.86 | 0.11 | | | 0.99 |
| H1O15 | | | | | | | | | | | | | 0.01 | | 0.86 | | 0.12 | 0.99 |
| H2O15a | | | | 0.10 | | | | 0.01 | | | | | | | 0.86 | | | 0.97 |
| H2O15b | | | 0.19 | | | | | | | | | | | | 0.86 | | | 1.05 |
| H1O16 | | | | | | 0.09 | | | | 0.02 | | | | | | 0.86 | | 0.97 |
| H2O16a | | | | | | | | | | 0.01 | | | | 0.11 | | 0.86 | | 0.98 |
| H2O16b | | | | 0.10 | | | | | | | | | | | | 0.86 | | 0.96 |
| H1O17 | | | 0.03 | | | | | | | | | | | 0.07 | | | 0.86 | 0.96 |
| H2O17 | | | | | | | 0.09 | 0.02 | | | | | | | | | 0.86 | 0.97 |
| $\Sigma BV_{Conf.a}$ | 1.92 | 1.86 | 2 | 2.84 | 1.97 | 2.07 | 1.8 | 2.23 | 1.87 | 2.22 | 1.84 | 2.13 | 1.96 | 1.9 | 1.92 | 2.08 | 1.96 | |
| $\Sigma BV_{Conf.b}$ | 1.93 | 1.842 | 2.21 | 1.97 | 1.97 | 2.02 | 1.8 | 2.22 | 1.86 | 2.05 | 1.83 | 2.1 | 1.98 | 1.95 | 1.94 | 1.86 | 2.03 | |
